# Predictive value of chromosome 18q11.2‐q12.1 loss for benefit from bevacizumab in metastatic colorectal cancer: A post hoc analysis of the randomized phase III‐trial AGITG‐MAX

**DOI:** 10.1002/ijc.34061

**Published:** 2022-05-23

**Authors:** Erik van Dijk, Erik van Werkhoven, Rebecca Asher, Jennifer K. Mooi, David Espinoza, Hendrik F. van Essen, Harm van Tinteren, Nicole C. T. van Grieken, Cornelis J. A. Punt, Niall C. Tebbutt, Bauke Ylstra

**Affiliations:** ^1^ Department of Pathology, Cancer Center Amsterdam, Amsterdam UMC VU University Medical Center Amsterdam The Netherlands; ^2^ Biometrics Department Erasmus Medical Center Rotterdam The Netherlands; ^3^ Department of Biostatistics, NHMRC Clinical Trials Centre University of Sydney Camperdown Australia; ^4^ Olivia Newton‐John Cancer Research Institute Heidelberg Australia; ^5^ Department of Medicine University of Melbourne Melbourne Australia; ^6^ Peter MacCallum Cancer Institute Melbourne Australia; ^7^ Trial and Datacenter Princess Máxima Center for Pediatric Oncology Utrecht The Netherlands; ^8^ Julius Centre for Health Sciences and Primary Care University Medical Centre Utrecht, Utrecht University Utrecht The Netherlands; ^9^ Department of Epidemiology University Medical Centre Utrecht, Utrecht University Utrecht The Netherlands; ^10^ Department of Medical Oncology Austin Health Heidelberg Australia; ^11^ Department of Surgery University of Melbourne Melbourne Australia

**Keywords:** anti‐VEGF monoclonal antibody, bevacizumab, chromosome 18q, metastatic colorectal cancer, predictive biomarker, randomized controlled trial

## Abstract

The VEGF‐A monoclonal antibody bevacizumab is currently recommended for first‐line treatment of all metastatic colorectal cancer (mCRC) patients. Cost‐benefit ratio and side‐effects however necessitate patient selection. A large retrospective yet nonrandomized study showed that patients with loss of chromosome 18q11.2‐q12.1 in the tumor and treated with bevacizumab have 3 months improved median progression‐free (PFS) and overall survival (OS) benefit compared to patients without this loss and/or treatment modality. Implementation for loss of chromosome 18q11.2‐q12.1 as a marker in clinical practice mandates evidence in a randomized controlled trial for bevacizumab. Of the trials with randomization of chemotherapy vs chemotherapy with bevacizumab, the AGITG‐MAX trial was the only one with tumor materials available. Chromosome 18q11.2‐q12.1 copy number status was measured for 256 AGITG‐MAX trial patients and correlated with PFS according to a predefined analysis plan with marker‐treatment interaction as the primary end‐point. Chromosome 18q11.2‐q12.1 losses were detected in 71% of patients (181/256) characteristic for mCRC. Consistent with the nonrandomized study, significant PFS benefit of bevacizumab was observed in patients with chromosome 18q11.2‐q12.1 loss (*P* = .009), and not in patients without 18q loss (*P* = .67). Although significance for marker‐treatment interaction was not reached (*P*_interaction_ = .28), hazard ratio and 95% confidence interval of this randomized cohort (HR_interaction_ = 0.72; 95% CI = 0.39‐1.32) shows striking overlap with the nonrandomized study cohorts (HR_interaction_ = 0.41; 95% CI = 0.32‐0.8) supported by a nonsignificant Cochrane *χ* ^2^ test (*P* = .11) for heterogeneity. We conclude that post hoc analysis of the AGITG‐MAX RCT provides supportive evidence for chromosome 18q11.2‐q12.1 as a predictive marker for bevacizumab in mCRC patients.

## Graphical abstract

What's new?

Survival among metastatic colorectal cancer (mCRC) patients can be significantly improved with the monoclonal antibody bevacizumab, though patient selection is needed in order to ensure favorable cost‐benefit ratio. A promising marker for this task is chromosome 18q11.2‐q12.1 loss. Here, the predictive capacity of 18q loss was evaluated in mCRC samples from bevacizumab‐treated patients enrolled in the AGITG‐MAX randomized trial. Data show that, compared to patients without 18q loss, those lacking 18q had better progression‐free survival following bevacizumab therapy. The study highlights the utility of 18q as a predictive marker for bevacizumab response and cost‐benefit assessment in mCRC patients.

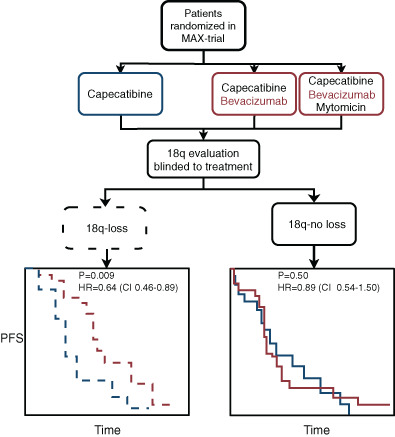

**Abbreviations**
CI: confidence interval
CMS: consensus molecular subtype
FFPE: formalin‐fixed and paraffin embedded
HR: hazard ratio
mCRC: metastatic colorectal cancer
ORR: overall response rate
OS: overall survival
PFS: progression free survival
RCT: randomized controlled trial
VEGF: vascular endothelial growth factor
WGS: whole genome sequencing

## 1 INTRODUCTION

Current guidelines for palliative treatment of metastatic colorectal cancer (mCRC) patients include treatment with chemotherapy combined with the VEGF‐inhibitor bevacizumab.^1^, ^2^ This recommendation is warranted by two large clinical trials where bevacizumab plus chemotherapy was compared to a chemotherapy only regimen.^3^ These trials showed a significant improvement in median survival of up to 3 months for bevacizumab supplementation to fluoropyrimidine based chemotherapy^4^, ^5^ and most benefit was obtained for a maintenance regimen.^6^, ^7^, ^8^ Bevacizumab however only benefits a subset of mCRC patients,^9^, ^10^ and has been associated with significant costs. Combined with an appreciable toxicity profile and a 1% increase in treatment related deaths^6^, ^11^, ^12^ this leads to a relatively unfavorable cost‐benefit ratio for quality adjusted life years.^13^, ^14^, ^15^ Consequently, and despite the current guidelines, not all eligible patients routinely receive bevacizumab in first‐line.^16^, ^17^, ^18^ A 10% to 15% reduction in treatment cost due to expiring patents and approval of biosimilars like Mvasi is not expected to change this situation.^19^, ^20^ In order to achieve more responsible expenditure of increasingly limited health care funds, selection of patients to obtain an improved cost‐benefit ratio is important, which is recognized as an urgent clinical need by the European Society for Medical Oncology (ESMO).^1^

A variety of markers for bevacizumab in mCRC were proposed by us and others, including VEGF isoforms, inflammasome protein NLRP1, neuropilin‐1 (NRP‐1) a co‐receptor for VEGF and Apelin which has an assumed VEGF‐like function.^21^, ^22^, ^23^, ^24^ KRAS/BRAF mutations status was considered, but not found to predictive for bevacizumab.^25^ Also the RNA profiling based consensus molecular subtype 2 (CMS2) was recognized as having predictive value with improved PFS with bevacizumab in the randomized AGITG‐MAX trial.^26^, ^27^, ^28^ Of the four CMS subtypes, CMS2 is the one with particularly high chromosomal “copy number load” which, based on data from the CAIRO2,^29^ MoMa^8^ and Angiopredict^30^ studies combined, was independently suggested as a predictive marker for bevacizumab.^9^, ^28^ None of these markers however have been validated in larger uniform and randomized series. The only marker validated in large and independent datasets is chromosome 18q11.2‐q12.1 copy number status.^10^, ^31^ Data derived from 616 patients receiving bevacizumab demonstrates a 2 month longer median PFS in patients with tumors with chromosome 18q11.2‐q12.1 loss compared to patients with tumors with no‐loss of chromosome 18q, or patients that did not receive bevacizumab.^10^ The chromosome 18q marker was, however, not validated in a randomized trial. Consequently, the predictive role of 18q loss remains uncertain. We aimed to evaluate marker‐treatment interaction of chromosome 18q11.2‐q12.1 loss in a randomized controlled trial (RCT) involving bevacizumab. Of the various large mCRC RCTs performed where first‐line bevacizumab supplementation to fluoropyrimidine showed significantly improved PFS benefit, only tumor tissues were available from the AGITG‐MAX RCT and for about 50% of the patients.^3^, ^4^, ^5^, ^27^ The AGITG‐MAX trial is a 3‐arm Phase III trial with 471 included patients randomized in a 1:1:1 ratio for treatment with either capecitabine monotherapy (arm A) or capecitabine and bevacizumab (arm B) or capecitabine, bevacizumab and mitomycin (arm C). Bevacizumab supplementation, with‐ or without mitomycin, significantly improved median PFS with 3 months, but no significant improvement was observed for overall survival (OS).^27^ PFS was the primary endpoint of the MAX trial and the nonrandomized cohort study^10^ and was used as a primary end‐point in our study. PFS is considered as the most appropriate end point for first line treatments, given the number of lines of therapy and types of effective subsequent treatment options for mCRC patients with substantial variability on OS. This challenges the use of OS as an endpoint to assess the benefit attributable to a single line of therapy.^32^

Of all available tumor samples, we determined chromosome 18q11.2‐q12.1 copy number aberrations by shallow whole genome sequencing (WGS) at the Cancer Center Amsterdam, blinded for the clinical variables.^27^, ^33^ To warrant a prospective‐like evaluation we developed a predefined analysis plan with PFS and biomarker‐treatment interaction of the four respective patient groups (marker positive‐ and negative patients, with‐ and without treatment) as a primary end‐point. Correlative analyses were performed at the clinical trial center (CTC) in Sydney.

## 2 MATERIALS AND METHODS

### 2.1 Patient samples

Formalin‐fixed and paraffin embedded (FFPE) tumor material was available for 262 of the 471 patients included in the AGITG‐MAX trial.^27^ For six patients the amount of tissue was insufficient or DNA quality requirements were not met.^26^ For N = 225 patients, sequencing was successfully performed for one biopsy, for N = 31 patients sequencing was successfully performed for two biopsies (Table S1). Of the 256 (54%) 18q‐evaluable patients, two patients did not receive protocol treatment. All FFPE materials of primary tumors or metastasis from patients of the MAX study were obtained at primary resection prior to treatment (Clinical trial: Name of the registry: This is a molecular sub‐study of MAX clinical trial; Trial registration number: NCT00294359; URL of trial registry record: https://clinicaltrials.gov/ct2/show/record/NCT00294359).

### 2.2 Copy number analysis

Tumor‐rich regions were demarcated on hematoxylin and eosin (H&E) stained sections and macrodissected using subsequent hematoxylin stained sections. DNA isolations were performed as previously described.^34^ While blinded to the clinical data, DNA samples were processed for genome‐wide high resolution copy number analysis by shallow WGS as described by Scheinin et al.^33^ Briefly, genomic DNA was fragmented using a Covaris ME220 (Covaris Inc, Woburn, Massachusetts). Subsequently, NGS libraries were processed with 100 ng DNA using the Applied Biosystems 5500 SOLiD fragment library enzyme module and the amplification module. Adapters were added with the 5500 SOLiD fragment library barcode adapter kit (Bioo Scientific, Austin, Texas). Libraries were sequenced with 50‐bp single‐read shallow whole‐genome sequencing (WGS) performed on a HiSeq 4000 (Illumina, San Diego, California). Sequence reads were aligned against the reference genome (GRCh37/hg19) with Burrows‐Wheeler Alignment tool (BWA aln; v0.5.9)^35^ and deduplicated with Picard tools (v1.61).^36^ Reads with mapping quality lower than 37 were excluded from further analysis. Samples (N = 7) with less than 1 million uniquely mapped nonduplicate reads were excluded. Copy number analysis was performed with QDNAseq (v1.12.0),^33^ NoWaves (v.0.6),^37^ DNAcopy (v1.50.1)^38^ and CGHcall (v2.38.0).^39^ Sequencing coverage and quality statistics for each sample are summarized in Table S1.

Chromosome 18q11.2‐q12.1 copy number status was determined for all samples as previously described.^10^ For the 31 patients that had DNA available for multiple biopsies, discordant 18q11.2‐q12.1 copy number calls were observed for N = 12 and assigned as “loss.” A per patient dichotomized list of chromosome 18q11.2‐q12.1 copy number status was transferred to the NHMRC Clinical Trials Centre (author R.A.) for statistical analysis (Table S1).

### 2.3 Power calculations

We calculated the sample size that would be required to analyze the interaction of chromosome 18q11.2‐q12.1 loss and bevacizumab within the 3‐arm MAX‐RCT with PFS as a primary end‐point. The power analysis was performed according to Schmoor et al,^40^ while assuming two‐thirds of samples come from patients treated with bevacizumab (CB and CBM trial arms combined) and one third without bevacizumab (C arm only). The incidence of 18q11.2‐q12.1 loss was assumed to be 70%, consistent with our previous results for mCRC.^10^, ^31^ The univariate hazard ratio (HR) of 0.63 for bevacizumab treatment was taken from the overall patient population of the AGITG‐MAX trial.^27^ Assuming no benefit in the 18q11.2‐q12.1 no‐loss group (equivalent to interaction HR_interaction_ = 0.43) the analysis with the evaluable tumor samples from 256 patients would have a power of 83% to detect a significant interaction at *P* = .05 (two‐sided test).

### 2.4 Statistical analysis

The analysis was performed according to an analysis plan that was finalized prior to any data processing. The primary outcome for our study was interaction of 18q status with PFS.^32^ Secondary outcomes were interaction of 18q status with OS and objective overall response (ORR).

All analyses were based on the intention‐to‐treat principle. Baseline demographics have been summarized by treatment group as frequency (%). Median PFS and OS were calculated using the method of Kaplan‐Meier.^41^ To assess the predictive value of the markers for chromosome 18q11.2‐q12.1, Cox proportional hazards models were used including a marker‐by‐treatment interaction term alongside the main effects of treatment and marker. The proportional‐hazards assumption of the Cox regression was verified by inspection of the Schoenfeld residuals. Multivariable analysis was conducted, adjusting the models for age (dichotomized at the median), number of metastases (1 or >1), prior adjuvant therapy and tumor side. To assess the predictive utility for ORR, odds ratios (OR) were calculated and a logistic regression model was used, incorporating the interaction term as described above. Results are presented alongside the corresponding 95% confidence interval (CI) and *P*‐value according to the log‐rank test. All analyses were conducted using SAS version 9.4 and plots were produced using Stata version 15.

## 3 RESULTS

### 3.1 Characteristics of the 256 AGTIG‐MAX 18q‐evaluable patients

The following baseline characteristics were evaluated: Eastern Cooperative Oncology Group (ECOG^42^) performance status, age, prior adjuvant therapy, side of disease, number of metastases, gender, extent of disease at baseline and KRAS/BRAF status. Baseline characteristics for our study are comparable to those that could not be evaluated for 18q loss (Table 1), although a slightly lower proportion of patients received adjuvant therapy (*P* = .07), or had local involvement of disease (*P* = .001), either or both of which may impact survival outcomes. For the 18q‐evaluable patients, median PFS was 8.0 months (95% CI = 7.0‐8.8) and median OS was 19.8 months (95% CI = 17.3‐22.2). For the overall AGTIG‐MAX trial population, survival was significantly shorter (PFS *P* = 0.001; HR = 0.73; 95% CI = 0.61‐0.89; OS *P* = .001; HR = 0.73; 95% CI = 0.61‐0.89; Figure S1A,B).

**TABLE 1:** Summary of baseline demographics of the 18q‐evaluable patients, compared to all patients in the AGITG‐MAX trial and patients not evaluable for copy numbers

	All patients	Excluded patients	18q evaluable patients	*P*‐value
	(*N* = 471)	(*N* = 215)	(*N* = 256)	
Treatment group				
C	156 (33%)	76 (35%)	80 (31%)	.38
B + M	315 (67%)	139 (65%)	176 (69%)	
ECOG status				
0	263 (56%)	114 (53%)	149 (58%)	.27
1/2	208 (44%)	101 (47%)	107 (42%)	
Age (years)				
≥67	244 (52%)	106 (49%)	138 (54%)	.35
Prior adjuvant therapy				
Yes	123 (26%)	65 (30%)	58 (23%)	.07
Side of disease in colon				
Right	124 (26%)	53 (25%)	71 (28%)	.32
Left	316 (67%)	144 (67%)	172 (67%)	
Unknown	31 (7%)	18 (8%)	13 (5%)	
Number metastases				
≤1	272 (58%)	122 (57%)	150 (59%)	.71
>1	199 (42%)	93 (43%)	106 (41%)	
Sex				
Male	295 (63%)	132 (61%)	163 (64%)	.63
Female	176 (37%)	83 (39%)	93 (36%)	
Extent of disease at baseline				
Local involvement	169 (36%)	94 (44%)	75 (29%)	.001
Liver involvement	353 (75%)	168 (78%)	185 (72%)	.17
Lung involvement	185 (39%)	80 (37%)	105 (41%)	.45
Bone involvement	18 (4%)	11 (5%)	7 (3%)	.23
Peritoneal involvement	84 (18%)	45 (21%)	39 (15%)	.12
Other involvement	49 (10%)	21 (10%)	28 (11%)	.76
KRAS				
Wild type	224 (71%)	44 (71%)	180 (71%)	>.95
Mutant	90 (29%)	18 (29%)	72 (29%)	
Not available	157	153	4	
BRAF				
Wild type	278 (11%)	55 (89%)	224 (89%)	>.95
Mutant	35 (89%)	7 (11%)	27 (11%)	
Not available	158	153	5	

*Note*: *P*‐values were calculated with a *χ* ^2^ test. ECOG performance status.^37^

Abbreviations: C, capecitabine monotherapy arm; B + M, capecitabine and bevacizumab with or without mitomycin.

Of the 18q‐evaluable patients 35% received capecitabine alone, and 65% capecitabine and bevacizumab with‐ or without mitomycin (Table S2). Chromosome 18q‐evaluable patients receiving capecitabine alone, had a significantly worse PFS compared to patients treated with bevacizumab with‐ or without mitomycin (*P* = .0097; HR = 0.69; 95% CI = 0.52‐0.92; Figure S1C). PFS benefit from bevacizumab of the chromosome 18q‐evaluable patients was less compared to that observed in both the entire AGITG‐MAX trial^27^ (HR = 0.61; 95% CI = 0.50‐0.74) and the collective nonrandomized study cohorts (HR = 0.64; 95% CI = 0.54‐0.75). OS benefit for bevacizumab was not observed within the AGITG‐MAX RCT,^27^ hence neither in the 18q‐evaluable selection of patients (*P* = .36; HR = 0.87; 95% CI = 0.63‐1.18, Figure S1D).

**FIGURE 1:**
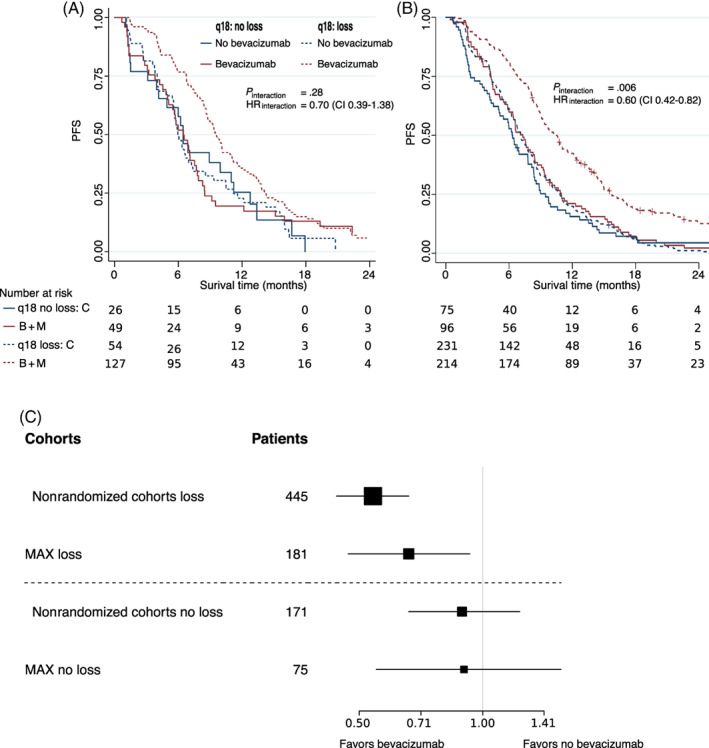
Progression free survival characteristics stratified by bevacizumab treatment and chromosome 18q11.2‐q12.1 copy number status. (A) Kaplan‐Meier analysis for the 18q‐evaluable AGITG‐MAX RCT cohort (N = 256); (B) Kaplan‐Meier analysis for the combined nonrandomized study cohorts (N = 616).^10^ Patients treated with capecitabine monotherapy (C, blue lines), with capecitabine and bevacizumab with‐ or without mitomycin (B + M, red lines), patients with chromosome 18q11.2‐q12.1 loss tumors (dashed lines), with no‐loss tumors (solid lines). Number of patients at risk below the *x*‐axis. (C) Forest plot of hazard ratio (HRs, black squares) and 95% confidence intervals (CIs), horizontal lines bevacizumab vs no‐bevacizumab patients. First column; patient selection, randomized (MAX) and nonrandomized^10^ by 18q11.2‐q12.1 status (loss and no loss); second column number of patients (#patients). [Correction added on 26 July 2022, after first online publication: Figure 1B y‐axis label has been changed from OS to PFS.] [Color figure can be viewed at wileyonlinelibrary.com]

The genome‐wide frequency of chromosomal copy number gains and losses for all 256 18q evaluable AGITG‐MAX patient samples was very similar to that observed previously for mCRC, with 71% (N = 181) chromosome 18q11.2‐q12.1 loss, which was 72% for the collective nonrandomized study cohorts (Figure S2).^10^, ^31^ Based on this frequency and randomization over the 3‐arms, chromosome 18q11.2‐q12 status was distributed across the four categories as expected, within the capecitabine monotherapy no‐loss category 10% (N = 26) and 21% in the chromosome 18q11.2‐q12.1 loss category (N = 54), in the bevacizumab supplemented no‐loss category 19% (N = 49) and 50% (N = 127) in the chromosome 18q11.2‐q12.1 loss category (*P* = .46; Table S2; Figure 1A). No correlations were found with the two most common side‐effects factors for bevacizumab treatment (hypertension, *P* = .85 and proteinuria, *P* = .64) and 18q‐11.2‐q12.1 loss (Table S3).

In summary, the 18q evaluable patient selection of N = 256 is genetically comparable to earlier studies. Both PFS and OS are marginally, but significantly longer compared to both the entire AGITG‐MAX trial cohort and the collective nonrandomized study cohorts, while bevacizumab benefit for the 18q‐evaluable patient cohort is reduced.

### 3.2 Predictive value of chromosome 18q11.2‐q12.1 copy number

Within the 71% (N = 181) of patients with chromosome 18q11.2‐q12.1 loss, a significant benefit from bevacizumab is observed (*P* = .009; HR = 0.64; 95% CI = 0.46‐0.89). No significant PFS benefit of bevacizumab is observed in the 29% (N = 75) no‐loss patients of which 19% (N = 49) received bevacizumab compared to 10% (N = 26) that did not receive bevacizumab (*P* = .67; HR = 0.89; 95% CI = 0.54‐1.50, Figure 1A). Hazard ratios and confidence intervals overlapped with those of the nonrandomized study (Figure 1B) where for the 72% (N = 445) of patients with chromosome 18q11.2‐q12.1 loss, a significant benefit from bevacizumab was observed (*P* < .001, HR = 0.54; 95% CI = 0.44‐0.66), whereas no significant PFS benefit of bevacizumab was observed in the 28% (N = 171) no‐loss patients (*P* = .50; HR = 0.89; 95% CI = 0.66‐1.23). The randomized MAX cohort thereby validates the association of chromosome 18q11.2‐q12.1 loss with survival of patients treated with bevacizumab.

Notwithstanding these striking similarities of PFS with our previous findings,^10^ in the much larger nonrandomized study (*P*_interaction_ = .006; HR_interaction_ = 0.6; 95% CI = 0.42‐0.82) (Figure 1A vs Figure 1B), significance for the predefined primary end‐point by PFS biomarker‐treatment interaction was not reached (*P*_interaction_ = .28; HR_interaction_ = 0.72; 95% CI = 0.39‐1.32). Multivariate analyses were done that incorporated the number of metastases (dichotomized as 1 vs 2 or more), age, previous adjuvant treatment and location of tumor (right side or left side). Results of the multivariate analyses were similar to those of the univariate analyses; with a significant bevacizumab benefit found for patients with chromosome 18q11.2‐q12.1 loss (*P* = .02; HR = 0.66; 95% CI = 0.47‐0.93) and no significant benefit for patients without 18q loss (*P* = .7; HR = 0.90; 95% CI = 0.51‐1.55). No statistical significance was reached for the interaction (*P*_interaction_ = .34; HR_interaction_ = 0.74; 95% CI = 0.39‐1.39). The fact that no statistical significance was reached for the interaction may be attributable to the observed effect size which was smaller than assumed for the power calculations (HR_interaction_ = 0.43).

This post hoc analysis was specifically prompted to evaluate the interaction observed in the retrospective nonrandomized cohort (*P*_interaction_ = .006; HR_interaction_ = 0.6; 95% CI = 0.42‐0.82^10^), in a randomized setting. Cochran's *Q* test^43^ showed no significant difference between the interaction observed in the randomized AGITG‐MAX 18q‐evaluable cohort and the nonrandomized study cohorts (*P* = .11; Figure 1C).

Benefit of bevacizumab on OS in the capecitabine monotherapy vs capecitabine in combination with bevacizumab, with‐ or without mitomycin was not significant for the entire AGITG MAX trial.^27^ Consequently, neither a significant OS benefit of bevacizumab treated patients with either 18q11.2‐q12.1 loss or no‐loss patients was reached accompanied by a negative marker treatment interaction of *P*_interaction_ > .95; HR = 1.01; 95% CI = 0.52‐1.96 (Figure S3). In line with earlier observations however, loss of chromosomal 18q was prognostic for OS, since patients with a loss of chromosome 18q11.2‐q12.1 had a significantly better OS,^44^, ^45^ independent of bevacizumab supplementation. In the AGITG‐MAX 18q‐evaluable cohort, response data was available for 238 patients. Similar to the results for OS, this cohort showed no significantly improved ORR for bevacizumab and therefore no significant improved response of bevacizumab treated patients regardless of 18q11.2‐q12.1 loss status was found, with a marker treatment interaction significance of *P*_interaction_ = .61; OR_interaction_ = 1.39; 95% CI = 0.39‐4.91.

### 3.3 Exploratory analyses

Two unplanned exploratory analyses were performed. First, recent results from Gambaro et al^18^ suggest that the marker may be valid in primary tumors only. For 31 of the 256 patients, multiple samples were available, and 12 of these were discordant between primary and metastasis and assigned “loss” in our analysis. We repeated the analysis for PFS excluding these 12 samples. This shows that these 12 samples did not affect the main conclusions for interaction (*P*_interaction_ = .29), significant PFS benefit for patients with loss of 18q11.1‐q12.1 (HR = 0.65; 95% CI = 0.46‐0.91) and not for patients with no‐loss of 18q11.2‐q12.1 (HR = 0.9; 95% CI = 0.54‐1.51). Second, the predictive effect of loss of entire chromosome 18q rather than 18q11.2‐q12.1 was also tested, which produced fewer patients with a loss (N = 115) and showed a nearly identical trend for interaction (*P*_interaction_ = .12), PFS benefit of bevacizumab for patients with loss of 18q11.1‐q12.1 (HR = 0.57; 95% CI = 0.38‐0.86) and not for patients with no‐loss of 18q11.2‐q12.1 (HR = 0.9; 95% CI = 0.60‐1.33).

## 4 DISCUSSION

Post hoc analysis of a cohort of N = 256 18q‐evaluable mCRC patients randomized for bevacizumab in the AGITG‐MAX trial provides supporting evidence for clinical relevance of chromosome 18q11.2‐q12.1 copy number status as a predictive marker. Significant benefit of bevacizumab was observed for patients with tumors that had chromosome 18q11.2‐q12.1 loss (*P* = .009) and not in the no‐loss patients (*P* = .67). By itself, the size of the AGITG‐MAX trial cohort fell short to obtain statistical significance by interaction analysis of chromosome 18q11.2‐q12.1 vs bevacizumab treatment (*P*_interaction_ = .28). We suspect that the nonsignificant interaction was most likely due to the smaller PFS benefit of bevacizumab in the AGITG‐MAX randomized trial which was furthermore reduced in the selection of patients for which tissues were available. We suspect that this reduced PFS benefit may be caused by a correlation between availability of tissues was and resectability of the primary tumor, which is supported by a slightly improved PFS for the 18q‐population. Despite the nonsignificant interaction, compared to the much larger nonrandomized validation study, the actual predictive value of the 18q‐11.2‐q12.1 marker is not significantly different as measured by Cochran's *Q*‐test (*P* = .11) nor by visual inspection of the KM‐plots (Figure 1). This comparative analyses supports that chromosome 18q11.2‐q12.1 no‐loss patients have reduced benefit of bevacizumab compared to patients with a loss.

Under the current guideline, where bevacizumab is recommended for all mCRC patients, withholding patient's bevacizumab would be regarded as unethical.^46^, ^47^ Yet, given the collective evidence presented, a prospective randomized trial may be proposed. If a noninferiority trial were to be considered, only marker negative patients would be included and bevacizumab withheld randomly to determine reduced benefit for *18q11.2‐q12.1* no‐loss patients. A noninferiority trial requires thousands of patients and is therefore time and cost consuming. Lack of consensus about the noninferiority margin, which defines statistical boundaries for what would be considered lack of benefit, would render the outcome debatable.^48^ An alternative would be a sufficiently powered prospective RCT, as retrospectively analyzed here, but still this would require withholding patient's bevacizumab and would require large numbers of patients.

A biological rationale between marker and drug supports clinical acceptance.^2^ However, understanding the observed effect on a molecular level remains challenging for two reasons: (a) The study of large copy number losses is hampered by a lack of functional genomics approaches.^49^ (b) Pinpointing genes or regulatory elements is complicated since the nonrandomized cohorts show that the entire chromosome *18q* is frequently lost in mCRC^31^ accompanied by a significant interaction with bevacizumab treatment and PFS.^10^ We found a similar trend in the results of this cohort. Thus, it may be possible that the entire chromosome *18q* loss supports the predictive effect. Hence, the observed effect may result from the contribution of multiple lost genes on chromosome *18q11.2‐q12.1* (N = 14 genes discussed previously^10^) or from the entire chromosome *18q* (N = 251 genes).

While clear guidelines exist for the approval of treatment modalities by government bodies, the criteria for approval of marker‐treatment combinations, particularly for existing treatments are more unclear. Although the most straightforward way towards approval is to demonstrate a significant treatment‐marker interaction, approval has been obtained commonly for markers with nonsignificant interaction *P*‐values or relied on justification with marker positive patients only.^50^, ^51^ In that light we reason that the presented evidence of loss of chromosome *18q11.2‐q12.1* as predictive marker is sufficient for clinical implementation in the situation where bevacizumab is not prescribed for all eligible patients due to financial restrictions. For chromosome *18q11.2‐q12* no‐loss patients with RAS (KRAS and NRAS)/BRAF wildtype tumors and left‐sided location of the primary tumor, anti‐EGFR in the first‐line is a viable alternative, now recommended solely beyond the first‐line.^29^

## 5 CONCLUSION

The analysis of a cohort of patients randomized for bevacizumab provides strong evidence for a predictive value of chromosome *18q11.2‐q12* copy number status. Particularly if financial restraints require patient stratification, loss of chromosome *18q11.2‐q12* diagnostics should be considered to select those patients that are predicted to have most benefit.

## AUTHOR CONTRIBUTIONS

Erik van Dijk, Erik van Werkhoven, Rebecca Asher, David Espinoza and Harm van Tinteren have been involved in the design of the work; the acquisition, analysis as well as interpretation of data and made substantial revisions to the article. Jennifer K. Mooi and Hendrik F. van Essen have performed laboratory experiments and data acquisition, Nicole C. T. van Grieken, Cornelis J. A. Punt, Niall C. Tebbutt and Bauke Ylstra have made substantial contributions to the conception and design of the work; drafted the work or substantively revised it. The work reported in the article has been performed by the authors, unless clearly specified in the text.

## CONFLICT OF INTEREST

The authors declare no conflicts of interest.

## ETHICS STATEMENT

Ethics approval was obtained from the Human Research Ethics Committee, Austin Health, centrally for all translational studies of the AGITG‐MAX trial. Only patients who provided prior written consent for their clinical data and tumor material are included in our study.

## Supporting information


**Appendix S1** Supporting Information.

**Table S1** Supporting Information.

## Data Availability

Sequence data has been deposited at the European Genome‐phenome Archive (EGA), which is hosted by the EBI and the CRG, under accession number EGAS00001005453.
