# Supplementary material for: Predictive value of chromosome 18q11.2‐q12.1 loss for benefit from bevacizumab in metastatic colorectal cancer: A post hoc analysis of the randomized phase III‐trial AGITG‐MAX
Source: Int J Cancer. 2022 May 23;151(7):1166–74. doi: 10.1002/ijc.34061 (PMC9545440; doi:10.1002/ijc.34061)
Supplement: Supplementary file 1 — Appendix S1 Supporting Information. [file IJC-151-1166-s002.pdf]

Predictive value of chromosome 18q11.2-q12.1 loss for benefit from bevacizumab in metastatic colorectal cancer; a post-hoc analysis of the randomized phase III-trial AGITG-MAX

Erik van Dijk, Erik van Werkhoven, Rebecca Asher, Jennifer K. Mooi[4], David Espinoza, Hendrik F. van Essen, Harm van Tinteren, Nicole C. T. van Grieken, Cornelis J. A. Punt, Niall C. Tebbutt, Bauke Ylstra

Supplementary Table S1 (Available separately as excel file)
Supplementary Table S2
Supplementary Table S3
Supplementary Figure S1
Supplementary Figure S2
Supplementary Figure S3

		C (n = 80)	B + M (n = 176)	P-value
Chromosome q18 loss	Yes	54 (68%)	127 (72%)	0.46
ECOG Status	0	44 (55%)	105 (60%)	0.50
	1+	36 (45%)	71 (40%)	
Prior Adjuvant Therapy	Yes	16 (20%)	42 (24%)	0.52
Age category	≥67	46 (58%)	92 (52%)	0.50
Side of disease	Right	21 (26%)	50 (28%)	0.94
	Left	55 (69%)	117 (66%)	
	Unknown	4 (5%)	9 (5%)	
Number metastases	≤1	47 (59%)	103 (59%)	>0.95
	>1	33 (41%)	73 (41%)	
Gender	Male	49 (61%)	114 (65%)	0.67
Extent of disease at baseline				
	Local Involvement	26 (33%)	49 (28%)	0.46
	Lung Involvement	56 (70%)	129 (73%)	0.65
	Lymph Node Involvement	38 (48%)	87 (49%)	0.79
	Lung Involvement	31 (39%)	74 (42%)	0.68
	Bone Involvement	3 (4%)	4 (2%)	0.68
	Peritoneal Involvement	16 (20%)	23 (13%)	0.19
	Other Involvement	7 (9%)	21 (12%)	0.52

Supplementary Table S2*: Distribution of Chromosome* 18q11.2-q12.1 *copy number status together with other baseline demographics across treatment arms (C) capecitabine monotherapy, (B + M) capecitabine and bevacizumab with or without mitomycin.*

**Side effect**	**Status**	**18q11.2-q12.1 loss**	**No 18q11.2-q12.1 loss**	***P*-value**
Hypertension	Hypertension	51	38	0.85
	No Hypertension	125	101	
Proteinuria	Proteinuria	69	59	0.64
	No Proteinuria	107	80	

Supplementary Table S3*: Distribution of side effects for bevacizumab for patients with tumors with and without* 18q11.2-q12.1 *loss. P-values were calculated with a chi-squared test.*

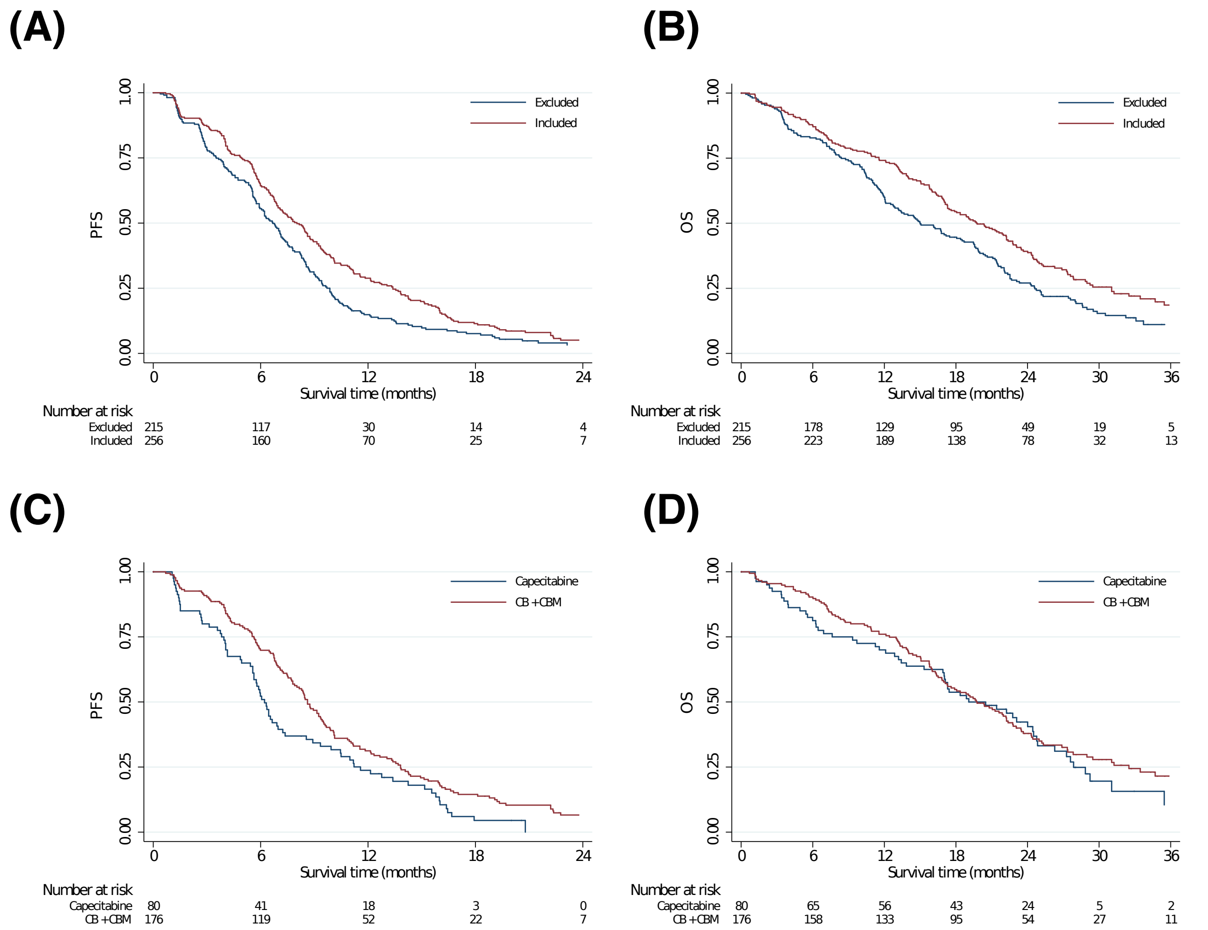

Supplementary Figure S1. Overall survival and Progression Free Survival characteristics of AGIGT-MAX RCT cohort. *Top, Kaplan-Meier analysis for (A) PFS and (B) OS of the 18q-evaluable (red) and non-evaluable (blue) cohort; Bottom, Kaplan-Meier analysis of the 18q-evaluable cohort for (C) PFS and (D) OS of patients treated with capecatibine (blue) and treated with capecatibine and bevacizumab with- or without mytomycin (red). Number of patients at risk below the x-axis.*

(A)

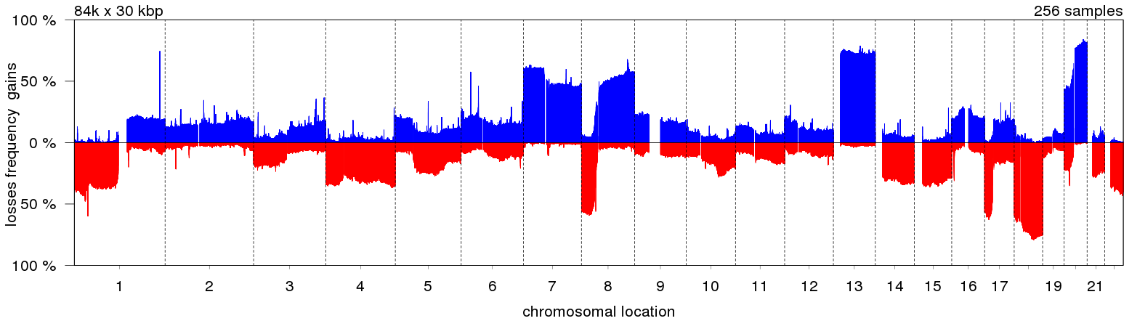

(B)

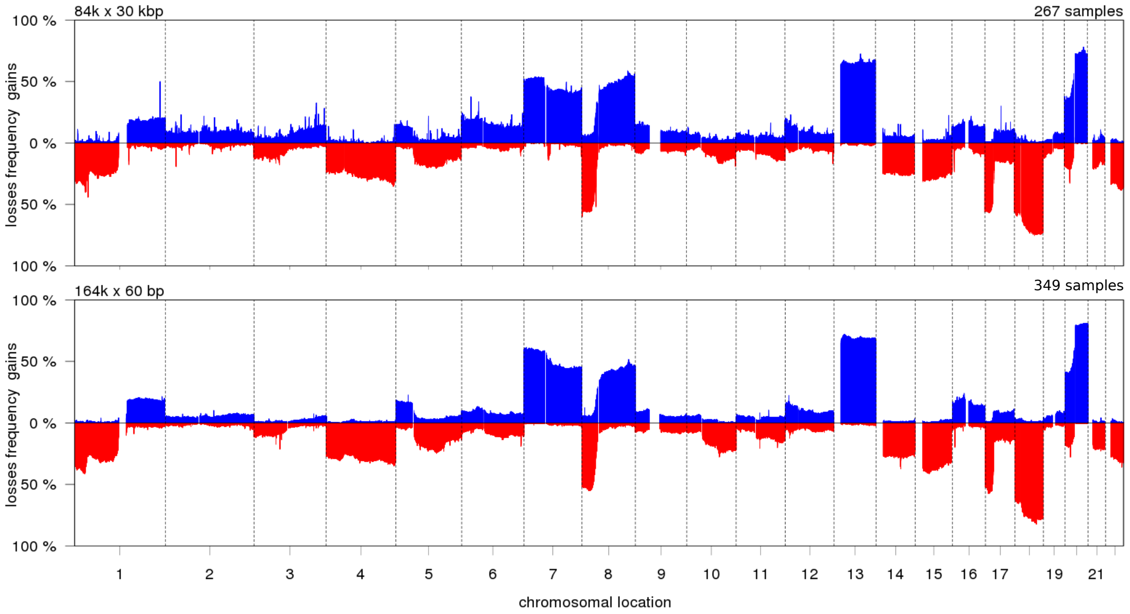

Supplementary Figure S2. Frequencies of chromosomal copy number aberrations. *(A) Frequency plot of AGITG-MAX RCT 18q-evaluable cohort (N=256, determined by WGS); (B) Frequency plots of non-randomized cohorts (n=616)* [10]*; Top panel, Frequency plot of tumor samples from non-randomized cohorts determined by WGS (n=267); (Bottom panel) Frequency plot of tumor samples from non-randomized cohorts determined by arrayCGH (n=349); x-axis, chromosomal bins sorted by chromosomal position; vertical dotted lines, boundaries between chromosomes. y-axis, percentage of tumors with gains (positive values) or losses (negative values). 84k x 30 kbp is, copy number calls for 84.000 bins of 30kbp each are plotted for WGS. 164k x 60 bp is, copy number calls for 164.000 probes of 60bp each are plotted for arrayCGH*

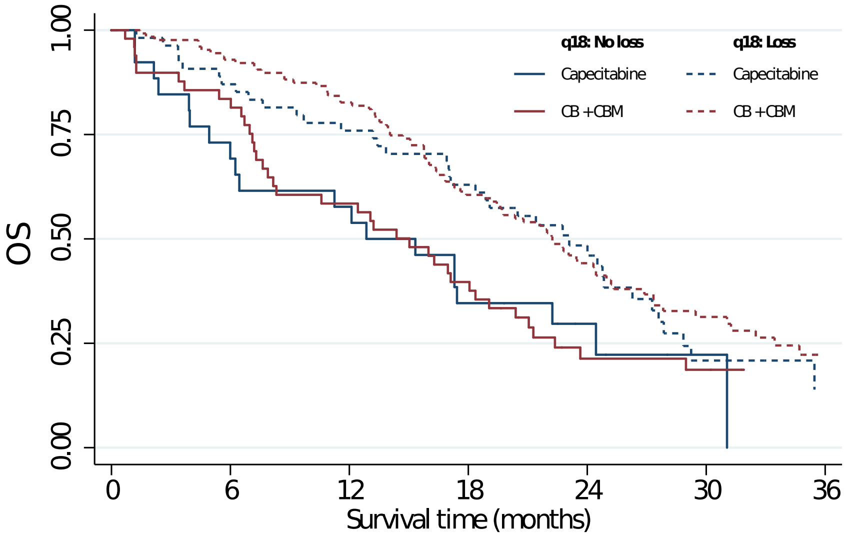

Number at risk

	0	6	12	18	24	30	36
q18 no loss: Capecitabine	26	18	15	9	4	1	0
CB +CBM	49	40	28	19	8	7	4
q18 loss: Capecitabine	54	47	41	34	20	4	2
CB +CBM	127	118	105	76	46	20	7

Supplementary Figure S3*. Overall Survival characteristics stratified by bevacizumab treatment and *chromosome 18q11.2-q12.1 copy number status*. *Kaplan-Meier analysis for the 18q-evaluable AGITG-MAX RCT cohort (N=256); Patients treated with capecitabine monotherapy (C, blue lines), with capecitabine and bevacizumab with- or without mitomycin (B + M, red lines), patients with chromosome 18q11.2-q12.1 loss tumors (dashed lines), with no-loss tumors (solid lines). Number of patients at risk below the x-axis.*
